# Genes Suggest Ancestral Colour Polymorphisms Are Shared across Morphologically Cryptic Species in Arctic Bumblebees

**DOI:** 10.1371/journal.pone.0144544

**Published:** 2015-12-10

**Authors:** Paul H. Williams, Alexandr M. Byvaltsev, Björn Cederberg, Mikhail V. Berezin, Frode Ødegaard, Claus Rasmussen, Leif L. Richardson, Jiaxing Huang, Cory S. Sheffield, Suzanne T. Williams

**Affiliations:** 1 Department of Life Sciences, The Natural History Museum, London, United Kingdom; 2 Department of General Biology and Ecology, Novosibirsk State University, Novosibirsk, Russia; 3 Swedish Species Information Centre, Swedish University of Agricultural Sciences, Uppsala, Sweden; 4 Entomological Department (Insectarium), The Moscow Zoo, Moscow, Russia; 5 Norwegian Institute for Nature Research, Trondheim, Norway; 6 Department of Bioscience, Aarhus University, Aarhus, Denmark; 7 Gund Institute for Ecological Economics, University of Vermont, Burlington, Vermont, United States of America; 8 Key Laboratory for Insect-Pollinator Biology of the Ministry of Agriculture, Institute of Apicultural Research, Chinese Academy of Agricultural Sciences, Beijing, China; 9 Royal Saskatchewan Museum, Regina, Saskatchewan, Canada; SOUTHWEST UNIVERSITY, CHINA

## Abstract

Our grasp of biodiversity is fine-tuned through the process of revisionary taxonomy. If species do exist in nature and can be discovered with available techniques, then we expect these revisions to converge on broadly shared interpretations of species. But for the primarily arctic bumblebees of the subgenus *Alpinobombus* of the genus *Bombus*, revisions by some of the most experienced specialists are unusual for bumblebees in that they have all reached different conclusions on the number of species present. Recent revisions based on skeletal morphology have concluded that there are from four to six species, while variation in colour pattern of the hair raised questions as to whether at least seven species might be present. Even more species are supported if we accept the recent move away from viewing species as morphotypes to viewing them instead as evolutionarily independent lineages (EILs) using data from genes. EILs are recognised here in practice from the gene coalescents that provide direct evidence for their evolutionary independence. We show from fitting both general mixed Yule/coalescent (GMYC) models and Poisson-tree-process (PTP) models to data for the mitochondrial COI gene that there is support for nine species in the subgenus *Alpinobombus*. Examination of the more slowly evolving nuclear PEPCK gene shows further support for a previously unrecognised taxon as a new species in northwestern North America. The three pairs of the most morphologically similar sister species are separated allopatrically and prevented from interbreeding by oceans. We also find that most of the species show multiple shared colour patterns, giving the appearance of mimicry among parts of the different species. However, reconstructing ancestral colour-pattern states shows that speciation is likely to have cut across widespread ancestral polymorphisms, without or largely without convergence. In the particular case of *Alpinobombus*, morphological, colour-pattern, and genetic groups show little agreement, which may help to explain the lack of agreement among previous taxonomic revisions.

## Introduction

Recently authors have sought to reduce subjectivity in the practice of species discovery or ‘delimitation’ [1] by comparing the results from applying multiple criteria in an explicitly integrative taxonomy [2]. This is a search for congruent results from multiple criteria as corroboration, even though the different sets of characters employed (from different disciplines) may have different evolutionary constraints requiring different evolutionary explanations [2]. Indeed, not all studies using different criteria can be expected to agree, because relationships between criteria and speciation events differ [1, 3, 4]. It has even been acknowledged that those criteria that relate most closely to speciation could form a minority pattern in some cases [2]. But for some arctic bumblebees, taxonomic revisions by experienced specialists have shown unusually poor agreement on the number of species, we suggest in part because of poor agreement among results from applying different criteria.

Bumblebees (genus *Bombus* Latreille) have been described as morphologically homogeneous compared with other bees [5]. It is therefore unsurprising that bumblebee species were diagnosed initially in terms of the striking colour patterns of the hair on the dorsum of the body [6]. But then, more than a century ago, it was realised that the groups supported by skeletal morphology are actually more clearly circumscribed, and that within these morphological species, bumblebees are highly variable in colour patterns [7–10]. This is supported by recent genetic studies [11–14], reaffirming the long-held view that bumblebee colour patterns do not necessarily diagnose species [15]. Detailed studies of bumblebee variation have led to the realisation that species can be cryptic in both morphology and colour pattern [16, 17], now confirmed by genetic studies [18, 19].

Particularly intriguing is the observation that bumblebee colour patterns are often impressively similar among species [8, 20–26]. In many cases, a single species may show different colour patterns in different geographical regions, in each of which they may resemble closely other species as members of regional colour-pattern groups [24, 27]. Several possible explanations have been proposed (reviewed in [26, 28]), including Müllerian mimicry [22, 29]. Recent advances in understanding evolutionary relationships have begun to clarify the chronology of the evolution of some of these resemblances [30], an important pre-requisite to discerning causes [31]. In cases of regional colour-pattern resemblance, the similar species are often only distantly related, so that the similarity has been interpreted as the result of evolutionary convergence [26]. We explore another possible explanation for the evolution of colour-pattern resemblance, as an alternative to convergence: that in some situations a polymorphism within an ancestral species may have been inherited in parallel by several descendent species.

The bumblebees of the subgenus *Alpinobombus* Skorikov are a small group of closely related species [32]. Previous taxonomic revisions (including global revisions and broader regional faunal revisions with global referencing) of *Alpinobombus* by some of the most experienced bumblebee specialists have relied on both morphology (especially the more variable characters of the male penis valve, volsella and gonostylus, and of the female oculo-malar area and hind tibia) and on the colour patterns of the hair and have all differed in their conclusions on the number of species present (Table 1). Several recent faunal lists have accepted as valid just the five more clearly morphologically-diagnosable species (interpreted in a broad sense, from the shape of the female head, male genitalia, etc.): *B*. *alpinus*, *B*. *polaris*, *B*. *balteatus*, *B*. *neoboreus*, and *B*. *hyperboreus* [10, 33–35]. But variation in colour pattern prompted some authors to question whether at least two more species might be added to this list: *B*. *pyrrhopygus* (but using the junior synonyms *B*. *diabolicus* Friese and *B*. *alpiniformis* Richards for bees with the Scandinavian colour pattern of this species) and *B*. *kirbiellus*, even though both lacked clearly discrete diagnostic morphological characters [36–38]. The colour patterns of *Alpinobombus* species have been described as being both variable within species while showing close similarities among species [36, 37, 39–43]. These species are unusual even among bumblebees [10, 44] for occupying extreme arctic or alpine habitats [40, 43, 45]. Therefore, at least in the far north, they co-occur with few or no other bumblebee species [36, 37, 43, 46], which simplifies the system for analysis if mimicry were involved.

**Table 1:** Lists and numbers of Species of the Subgenus *Alpinobombus* from previous taxonomic revisions.^a^

	Richards 1931 [39]	Skorikov 1937 [43]	Milliron 1973 [36]	Løken 1973 [37]	This study
	*alpinus*	*alpinus*	*alpinus*	*alpinus*	*alpinus*
					*pyrrhopygus*
	*arcticus*	*arcticus*	*polaris*	*arcticus*	*polaris*
	*kincaidii*	*kincaidi*			
	*balteatus*	*balteatus*	*balteatus*	*balteatus*	*balteatus*
		*tristis*			
			*kirbyellus*		*kirbiellus*
	*neoboreus*	*neoboreus*		?	*neoboreus*
	*strenuus*	*strenuus*	*strenuus*	?	
					unnamed
					*natvigi*
	*hyperboreus*	*hyperboreus*	*hyperboreus*	*hyperboreus*	*hyperboreus*
Total	7	8	6	4	9
Split ^b^	2	3	0	0	-
Lumped ^b^	3	3	2	3	-

^a^Species names and spellings are those used in the original publications. The revision by Løken did not cover the New World fauna and so did not treat *B*. *neoboreus / B*. *strenuus*.

^b^Counts of split and lumped species compared to this study exclude the ‘unnamed’ taxon that we treat (conservatively) as unsampled previously.

Authors of the taxon names: *alpinus* (Linnaeus), *arcticus* Kirby, *balteatus* Dahlbom, *hyperboreus* Schönherr, *kincaidii* Cockerell, *kirbiellus* Dahlbom, *natvigi* Richards, *neoboreus* Sladen, *polaris* Curtis, *pyrrhopygus* Friese, *strenuus* Cresson, *tristis* Friese.

We use sequences for parts of the COI and PEPCK genes from freshly collected specimens and from museum specimens to recognise the evolutionarily independent lineages of the subgenus *Alpinobombus* as species and to estimate the phylogenetic relationships among these species. We then test whether the colour-pattern polymorphisms are more likely either to be ancestral, or to have arisen more recently and convergently within the daughter species. From our analyses we conclude: (1) that coalescents for the COI gene support nearly twice as many species (nine cf. five) as have been recognised from skeletal morphology and that three of these species, including one taxon not recognised previously, are also supported by PEPCK polymorphisms; and (2) that similarity in colour pattern among parts of these species is likely to have arisen from broadly conserved ancestral polymorphisms, not from convergence. The work reported here builds on a survey of the morphology and colour patterns of the North American *Alpinobombus* [42] and is part of a revisionary study of *Alpinobombus* bumblebees world-wide, which will treat type specimens and nomenclatural issues separately.

## Materials and Methods

### Ethics statement

No humans or vertebrate animals were used in this research. Field permits: (Russia) Bumblebees of the subgenus *Alpinobombus* are not included in any Red Lists and are not listed under the law ‘On environmental protection’ in Russia. None of the areas where field work was carried out are private. Only Meduza Bay in West Taimyr is a part of the Great Arctic Reserve, where the research was conducted with the permission of the Reserve management. Specimens from the research collection of Novosibirsk passed the State veterinary control before export on loan.

(Norway) No permits were needed for collecting of the Norwegian data.

(Sweden) None of the bees were collected within National Parks or Nature Reserves. They are not subject to any collection restrictions and therefore permits are not required. At present there is no legislation or restrictions that regulates the export of genetic samples from Sweden. There is no other restriction on the transfer of scientific material of this sort between EU member states.

(Denmark / Greenland) The Greenland Ecosystem Monitoring Coordination Group at the National Environmental Research Institute, Aarhus University, approved our research proposals for access and research activities in both 2010 and 2011.

(Canada) No permits were required for collection sites visited.

(USA) Collections from the Rocky Mountain Biological Laboratory in Colorado were made according to an agreement between the RMBL and the United States Forest Service.

**Fig 1:**
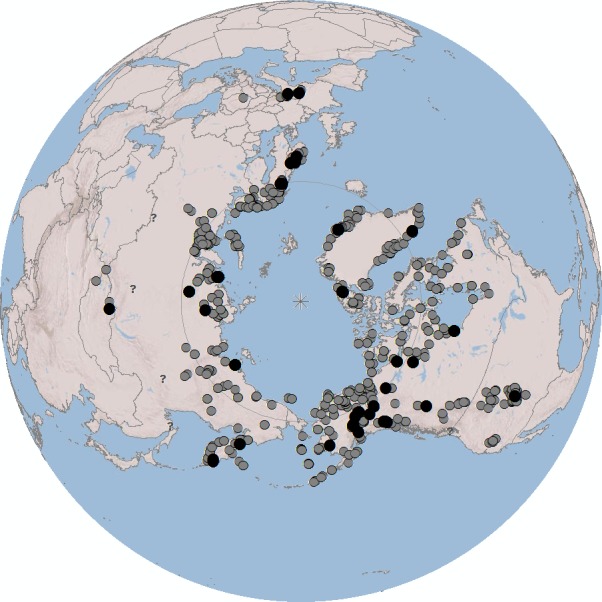
Global Sampling of the Subgenus *Alpinobombus*. Grey spots show sample sites for the 4345 specimens examined (*Alpinobombus* species are unknown from the tropics or from the southern hemisphere; collections listed in Table A in S1 File); black spots show the sites for the 173 samples with COI barcodes; and question marks show the sites for doubtful records (specimens supposedly from sites that combine low latitude with low elevation). Polar projection, North Pole (starred) at the centre of the map. Terrestrial national boundaries and the Arctic Circle are shown as narrow grey lines. Image created in ArcGIS using World_Shaded_Relief basemap which is Copyright 2014 ESRI.

We bring together samples of taxa of the subgenus *Alpinobombus* from throughout their global distributions. Taxonomic and geographical breadth is essential in a taxonomic revision for achieving a representative sample in order to assess the full range of patterns of variation and relationship [47, 48]. This is especially important for reducing the bias that might otherwise result if sampling were restricted to just one or two isolated regions or were to exclude major lineages. To cover all variation, we made a preliminary survey of specimens from museum collections (Fig 1; see the supporting information).

To recognize species of the subgenus *Alpinobombus*, we accept the broadly held concept of species as evolutionarily independent lineages (EILs) [1]. To recognize these species in practice, we use statistical tests designed to identify gene coalescents, which provide direct evidence for EILs [49]. Coalescence methods are suggested to be especially useful compared to less direct indicators (including criteria related to mate recognition and reproductive isolation) because they can detect lineage separation even in its early stages [1, 3]. We use one of the fastest evolving, most highly variable mitochondrial genes, coding for COI (cytochrome oxidase subunit I). This is particularly appropriate for detecting species because of its rapid coalescence within lineages [3, 50]. From among recently collected specimens showing the most obvious morphological and colour variation, we sequenced the ‘barcode’ region of the COI gene (Fig 1).

Most of the extraction, amplification, and sequencing work was done at the Biodiversity Institute of Ontario, Guelph, using standard protocols [51] and primers [52]. A minority of specimens were processed at the Institute of Apicultural Research, Beijing. Because the geographic range occupied by *Alpinobombus* is large and many areas are difficult to access, mitochondrial DNA with many copies per cell has the advantage of being more easily extracted, so that even pinned museum specimens can be sampled. Although 70% of *Alpinobombus* specimens surveyed are known to be less than a century old, only 8.6% are less than 10 years old. A problem with older museum specimens is that the sequences obtained are often not of full length [53]. Sequence alignments were made using the ClustalW function in MEGA (version 6.06, accessed 2014: megasoftware.net [54]) and amino acid translations were checked with EMBOSS Transeq (accessed 2014: ebi.ac.uk/Tools/st/emboss_transeq/).

To check for possible mismatches between trees for different genes, and hence for possible mismatches between gene trees and species trees [49, 55], we also sequenced the nuclear gene PEPCK (phosphoenolpyruvate carboxykinase; accession numbers for sequences listed in Table A in S2 File, obtained using primers from [32]). This gene has been popular for studies of bumblebees [11, 30, 32].

To recognise species, single-threshold general mixed Yule/coalescent (GMYC) models seek a transition expected in the tree-branching rates between interspecific branches and intraspecific branches [56–61]. GMYC analysis requires an ultrametric estimate of the phylogenetic tree that includes only unique haplotypes [59, 62] (T. Barraclough, pers. comm.) in order to avoid spurious inflation of estimates of terminal branching rates. After sorting the sequences by decreasing length, the longest examples of each unique haplotype are recognised using Collapse (version 1.2, accessed 2011: softpedia.com/get/Science-CAD/Collapse.shtml), which ignores sites with missing data. As outgroups for rooting the tree with this fast gene, we include species from closely related bumblebee subgenera [32]: *B*. (*Cullumanobombus*) *rufocinctus* Cresson, *B*. (*Pyrobombus*) *vagans* Smith, *B*. (*Bombus*) *ignitus* Smith, *B*. (*Bombus*) *terrestris* (Linnaeus), and *B*. (*Bombus*) *cryptarum* (Fabricius). The best nucleotide-substitution model for COI according to the Bayesian information criterion (BIC) obtained from MEGA is the general time-reversible model with a gamma-frequency distribution of changes among sites (GTR+Γ). BEAST (version 1.8.0, accessed 2013: beast.bio.ed.ac.uk) was used for Bayesian analysis of multiple model trees [63]. The clock model was set to the uncorrelated lognormal (relaxed clock), the tree-speciation prior was set to a constant-size coalescent process (consistent with the null hypothesis that there is a single species in the data), and the chain length for the Markov-chain Monte Carlo (MCMC) algorithm was set to two billion generations, with sampling of the trees every 200,000 generations. The sample of resulting trees from the MCMC algorithm was examined using Tracer (version 1.6.0, accessed 2013 [63]), which showed that stationarity had been achieved within the first 1% of the total MCMC generations. The large number of MCMC generations was needed with our data to increase effective sample sizes. A maximum clade-credibility tree was obtained from the post burn-in tree sample after rejecting the first 1% of sampled trees using TreeAnnotator (version 1.8.0, as for BEAST). After removal of the outgroups, the GMYC model was applied to the tree using the SPLITS library (version 1.0–11, accessed 2011: r-forge.r-project.org/projects/splits/) running on the R platform (version 2.12.1, accessed 2011: www.r-project.org). The oldest available name [64] is applied to each coalescent group as a prospective species. Multiple runs of all analyses were made and the stability of the results checked.

To check the robustness of the GMYC results by taking into account the uncertainty in estimates of phylogeny, we applied a Bayesian GMYC analysis [59] using the bGMYC library (version 1.0.2, accessed 2015: sites.google.com/site/noahmreid/home/software) on the R platform. bGMYC was applied to the last 100 trees from the sample of 10,000 trees from the BEAST run of two billion MCMC generations (a sample representing the last 20 million trees) that had been checked previously with Tracer for stationarity. bGMYC was run with the default settings (MCMC 50,000 generations, burn-in 40,000, thinning 100).

As another check on the number of species recognised, we apply another related approach for discovering species designed for use with single-locus data, looking for changes in the numbers of nucleotide changes along branches from between- to within-species relationships, based on Poisson-tree processes (PTP: [65]). PTP analysis is less demanding of tree information than the GMYC approach and there is some evidence that it can perform better [65]. We apply the Bayesian implementation on the bPTP server (accessed 2015: species.h-its.org) to a metric maximum-clade-credibility tree from COI barcodes obtained with MrBayes (version 3.1.2, accessed 2011: [66]), using the same data, outgroups, and nucleotide-substitution model, four chains (temperature 0.2), and 100 million generations of the MCMC algorithm with a 1% burn-in selected using Tracer. We used the default bPTP options after removing outgroups from the rooted tree.

The variation in colour patterns for the subgenus *Alpinobombus* includes many subtle differences [42], which are similar in the two sexes. In all cases colour patterns refer to the colour of the hair on various body regions, not to the colour of the body surface, which is brown or nearly black throughout. Generally, we observe that the presence of non-black hair on the different body segments is not independent, but appears to be conditionally dependent among segments [8] (e.g. there are no *Alpinobombus* bees with the hair on metasomal tergum 2 black and on tergum 3 yellow, or on tergum 4 orange and on tergum 5 black, although in both cases the reverse conditions are common). Therefore it should be reasonable to begin by coding bumblebee colour patterns for the presence or absence of areas of pale hair and then later adding characters as modifiers for the extent of the pale hair [26, 67]. As a first approximation, we assume that *Alpinobombus* colour patterns can be reduced to a representation as two principal characters, each with two states. Character (1): whether there are transverse yellow bands in the hair on the body, usually anteriorly and often posteriorly on the thoracic dorsum and/or on metasomal terga 1–2 (state B, banded: minimally with an obvious yellow band on metasomal tergum 2, varying from pale straw yellow to darker orange brown), or whether the hair in these areas is black or nearly black (state U, unbanded). Character (2): whether there is obvious pale hair (most often red or orange, but sometimes yellow or white) on the ‘tail’, consisting of metasomal terga 4–6 (state P, pale: minimally covering tergum 5 or 6), or whether the hair of the tail is black or nearly black (state D, dark). The most widespread colour pattern both among all bumblebees [26] and among the sister-group to the subgenus *Alpinobombus*, the subgenus *Bombus sensu stricto* [19], has both the yellow bands and a pale tail. Replacement of the pale bands and the pale tail with black can also be polymorphic within some *Bombus s*. *str*. species, although this is relatively rare (occurring in 3/17 and 1/17 species for the two characters respectively). Colour characters were scored for a sample of 1646 *Alpinobombus* specimens of both sexes from across the distribution range. To reconstruct the history of colour-pattern characters, we estimate the species tree using BEAST linked trees from COI and PEPCK exon and intron sequences. The best (unlinked) nucleotide-substitution models according to the MEGA BIC are HKY for both the PEPCK exon and introns, the tree-speciation prior was set to a birth-death process appropriate for a multi-species tree, and from examination of the results with Tracer, the MCMC algorithm was set to run for 1.5 billion generations. Outgroups and other settings were as for the first analysis. No fossils of *Alpinobombus* species are known for dating parts of the tree, so the phylogeny was calibrated with a date from a molecular study: Hines [68] estimated the age of divergence between the subgenus *Alpinobombus* and the subgenus *Bombus s*. *str*. to be 13 Ma. Mesquite (version 3.02, accessed 2015: mesquiteproject.org [69]) was used to reconstruct ancestral characters states on this tree. We used parsimony analysis because we wanted to retain information on alternative explanations rather than just the most likely. We coded polymorphic populations as a third intermediate ordered state, allowing state changes in either direction ([70]; W. Maddison, pers. comm.).

## Results

**Fig 2:**
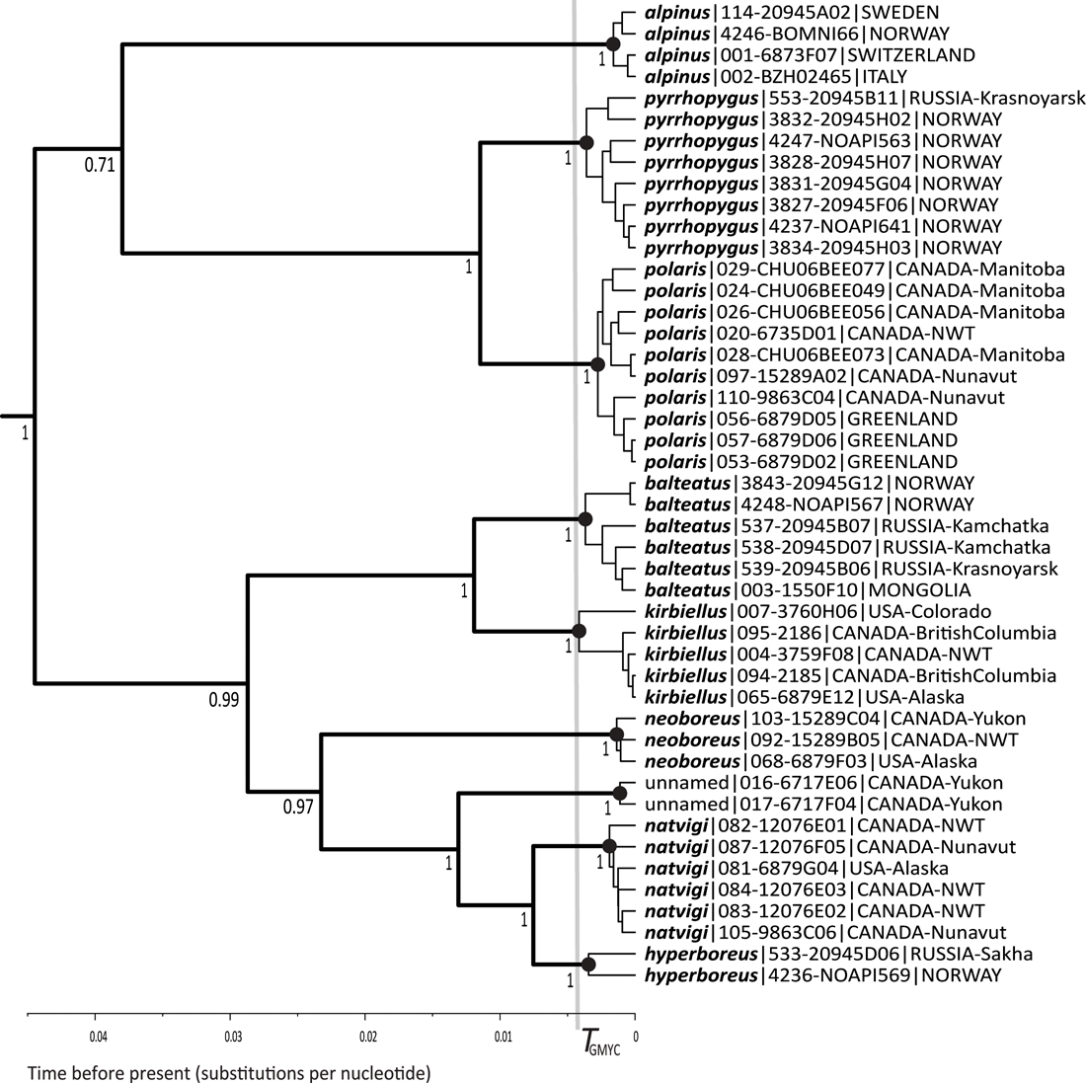
Recognising Species of the Subgenus *Alpinobombus* with GMYC. The 173 sequences reduced to 46 unique COI-barcode haplotypes on a BEAST ultrametric gene tree (outgroups not shown). The single threshold (*T* = -0.0041) from the GMYC model is shown by the vertical grey bar and the intersecting lineages are interpreted as subtending nine prospective species (black spots show the coalescent node for each species). The tree is the Bayesian ultrametric maximum-clade-credibility tree from a sample of trees after 1% burn-in from 2 billion generations of the MCMC algorithm in BEAST. Values next to the nodes are Bayesian posterior probabilities showing branch support. Each haplotype is represented by one of the longest sample sequences, labelled with a species name, a code that consists of an identifier from the project database and (after the hyphen) from the BOLD database, followed by its geographic origin.

We found 46 unique haplotypes among 173 COI-barcode samples of the subgenus *Alpinobombus* (accession numbers listed in Table A in S2 File). These COI data are information rich, with 92/658 nucleotide sites phylogenetically informative. The GMYC models show a significant change in branching rate through time in the COI barcode tree (likelihood ratio 14.03 between the GMYC multiple-species model and the null model that there is a single species in the group, *p* = 0.0028). This threshold (at -0.0041 substitutions per nucleotide) leads us to recognise nine candidates for prospective species (with a 95% confidence interval of 8–11 species) within *Alpinobombus* as in Fig 2, which also shows that each of these species is strongly supported as a monophyletic group.

**Fig 3:**
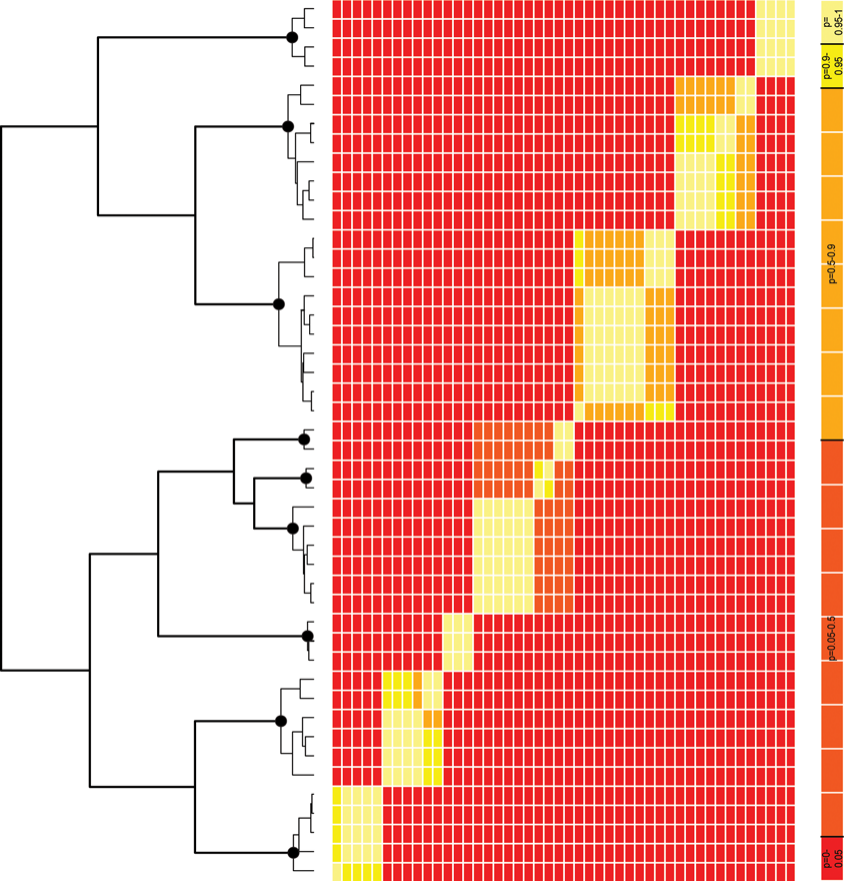
Recognising Species of the Subgenus *Alpinobombus* with Bayesian GMYC. The 173 sequences reduced to 46 unique COI-barcode haplotypes on one BEAST ultrametric gene tree (outgroups not shown). The posterior probability distribution (right, colour scale far right) is plotted against a sample tree from BEAST (left) to provide a ‘heat’ map of the probabilities that haplotypes are conspecific by bGMYC. Black spots show the coalescent node for each species from Fig 2.

The bGMYC analysis showed stationarity and a high modal coalescent/Yule ratio consistent with success. The posterior probability distribution is shown against the first sample tree in Fig 3 to provide a ‘heat’ map of the probabilities that the haplotypes are conspecific. Adopting a threshold probability of 0.5 from the posterior mean from the analysis (corresponding to a moderate position, midway between a ‘splitter’ and a ‘lumper’) recognises nine species within the subgenus *Alpinobombus* on the diagonal (unsurprisingly, the species with more haplotypes have more structure within them). These are the same nine species as are identified in the GMYC analysis in Fig 2.

**Fig 4:**
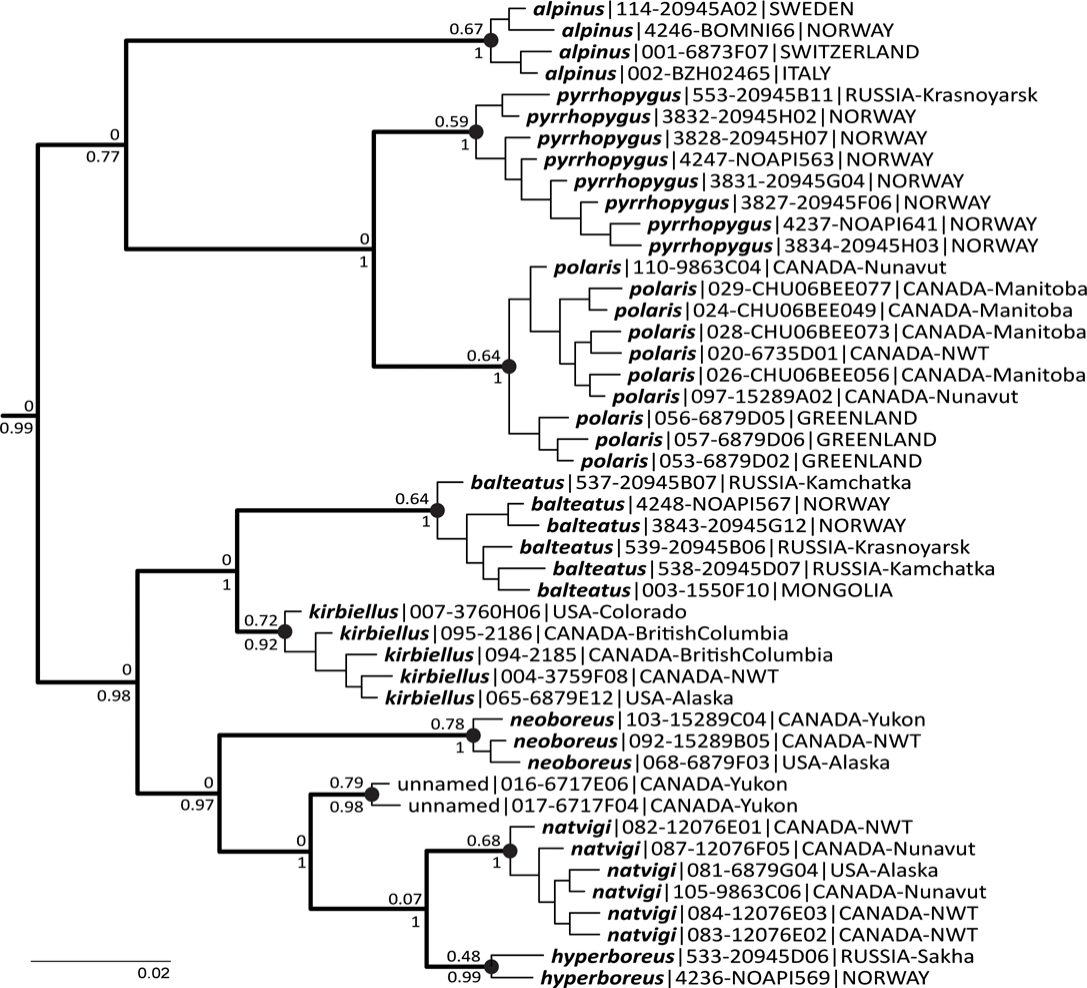
Recognising Species of the Subgenus *Alpinobombus* with Bayesian PTP. The 46 unique COI-barcode haplotypes as in Fig 2 on a MrBayes metric gene tree with the Bayesian PTP solution with highest support, showing nine prospective species (black spots show the coalescent node for each species; outgroups not shown). The tree is the maximum-clade-credibility tree after 1% burn-in from 100 million generations of the MCMC algorithm in MrBayes. Values above the nodes are PTP Bayesian support values that all daughter haplotypes belong to a single species population; values below the nodes are Bayesian posterior probabilities showing branch support. Haplotype selection and labels as in Fig 2. The scale bar for branch lengths shows 0.02 substitutions per base position.

The Bayesian PTP solution with the most support (Fig 4) recognises the same nine prospective species (with a range of 7–30 species), although the individual Bayesian support values for the species are not high (0.48–0.79).

**Fig 5:**
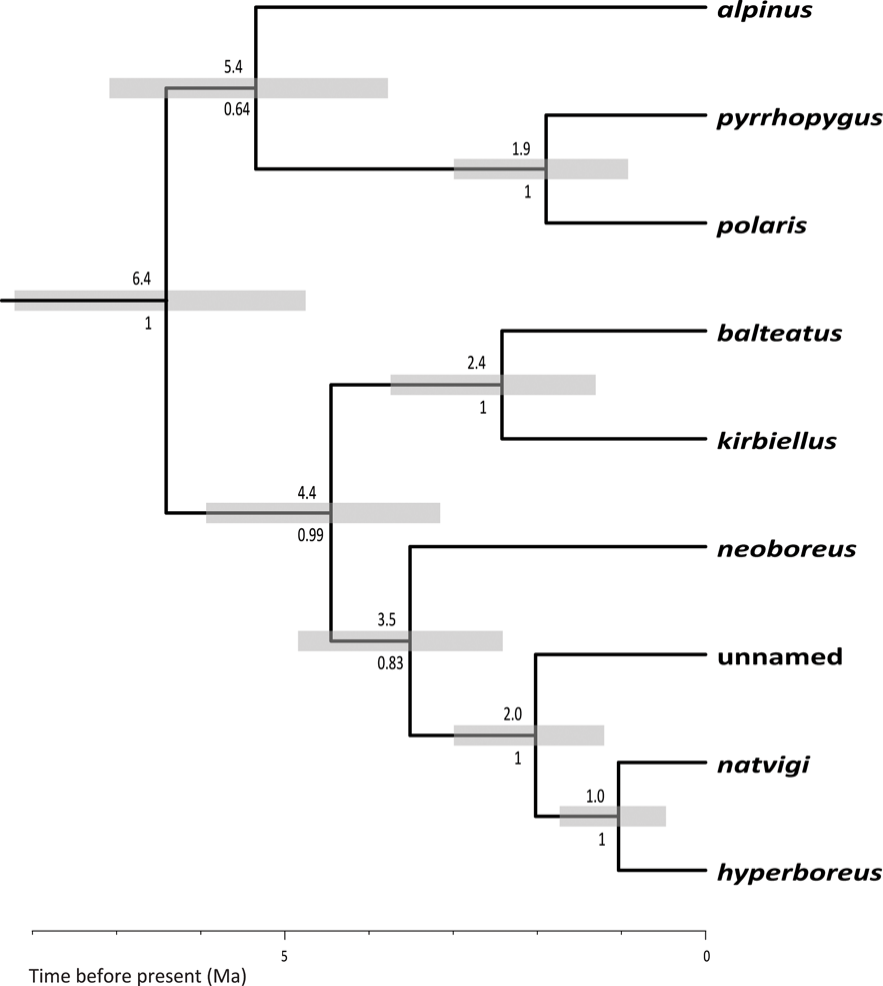
Estimate of phylogeny for species of the subgenus *Alpinobombus*. Estimated using a linked-tree BEAST analysis of COI-barcode and PEPCK exon and intron sequences for each species. Values below the nodes are Bayesian posterior probabilities showing branch support. Numbers above the nodes show the estimated dates of divergence events in Ma (millions of years before the present) calibrated with a molecular estimate for the date of divergence between the subgenus *Alpinobombus* and the subgenus *Bombus s*. *str*. from Hines (2008) and grey bars show the 95% confidence limits on the estimated dates of divergence.

As expected, we found much less variation in the PEPCK gene among species of the subgenus *Alpinobombus*. Only 4/903 nucleotide sites in our sequences are uniquely diagnostic for species, with unique PEPCK nucleotide changes supporting the species *B*. *alpinus* (base position 351), *B*. *neoboreus* (bp 755), and two for the unnamed species (bp 360, 533) (Table 2). Additional PEPCK nucleotide differences give further support for the distinction between the species pair *B*. *neoboreus /* unnamed (bp 769). The estimate of phylogeny for *Alpinobombus* species from the combined gene data is shown in Fig 5. This shows strong support for all groups except for the position of *B*. *alpinus*, which remains uncertain.

**Table 2:** PEPCK polymorphisms.^a^

Species	88	96	114	187	223	309	310	347	351	360	467	504	533	546	548	621	626	732	741	755	762	763	764	769	794	801	803	814	842	884	902
*alpinus* (2)	T	A	T	G	G	T	C	G	**G**	**C**	C	G	**G**	C	G	A	T	G/A	G	**C**	A	A	G	**T**	G	T	T	A	A	G	G
*pyrrhopygus* (3)	.	.	.	.	.	.	.	.	**A**	.	.	A	.	.	.	.	.	G	.	.	.	.	.	.	.	.	.	.	.	G/T	.
*polaris* (2)	.	.	.	G/A	.	.	.	.	**A**	.	C/T	G/A	.	.	.	.	.	.	.	.	.	.	.	.	.	.	.	.	.	.	.
*balteatus* (3)	.	.	T/G	.	G/A	T/A	.	.	**A**	.	.	.	.	C/A	.	.	T/G	.	.	.	.	.	.	.	.	T/G	T/A	.	.	.	.
*kirbiellus* (3)	.	.	.	.	.	.	.	G/A	**A**	.	.	.	.	.	G/A	.	.	.	.	.	.	.	.	.	.	.	.	A/G	.	.	.
*neoboreus* (2)	.	.	.	.	.	.	.	.	**A**	.	.	.	.	.	.	.	.	.	.	**A**	.	.	.	.	A	.	.	.	.	.	.
unnamed (2)	.	.	.	.	.	.	C/G	.	**A**	**A**	.	.	**T**	.	G/A	A/G	.	.	G/A	.	A/G	A/G	G/C	**A**	G/A	.	.	A/G	A/G	.	G/C
*natvigi* (2)	T/C	A/T	.	.	.	.	.	.	**A**	.	C/T	.	.	.	.	.	.	.	.	.	.	.	.	**A**	.	.	.	.	.	.	.
*hyperboreus* (2)	.	.	.	.	.	.	.	.	**A**	.	T	.	.	.	.	.	.	.	.	.	.	.	.	**A**	.	.	.	.	.	.	.

^a^ Numbers in the top row refer to nucleotide positions within a condensed alignment of the sequences with minimal gaps (903 base pairs), letters are FASTA codes for nucleotides but with additional polymorphisms shown explicitly. Dots indicate a nucleotide matching the first (or for position 732, the second) sequence. Species-diagnostic sites (positions 351 360 533 755 769) are shown in bold, and other polymorphic sites are shown without bold. Numbers next to species’ names are the numbers of sequences examined.

**Fig 6:**
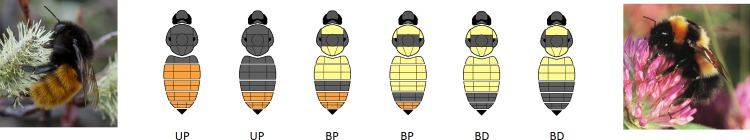
Colour Patterns of the Subgenus *Alpinobombus*. Simplified diagrams [26] representing the principal variation in colour patterns of the hair on the female dorsum coded as two two-state characters: UP, unbanded pale tail; BP, banded pale tail; BD, banded dark tail; with hair colours: yellow, yellow or yellow-brown hair; orange, orange-red or white hair; dark grey, black hair. Only female patterns are shown because females make up the majority of samples for social bumblebees, although male patterns are similar. Photos show: (left) *B*. *alpinus* and (right) *B*. *hyperboreus*. Photos by (left) A. Staverløkk and (right) G. Holmström.

**Fig 7:**
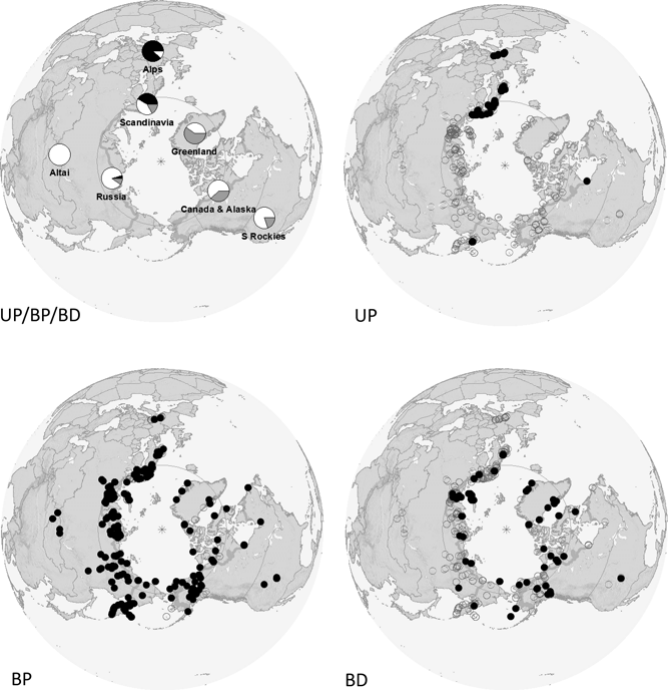
Distribution of Colour Patterns of the Subgenus *Alpinobombus*. Pie diagrams (UP/BP/BD) show relative frequencies of colour patterns (black, UP; white, BP; grey, BD) from a sample of 1646 colour-coded specimens with coordinates by regions (clockwise from the top): Greenland (*n* = 27), Canada and Alaska (*n* = 257), southern Rockies (*n* = 25), Russia excluding Murmansk Province (Kola Peninsula) (*n* = 654), Altai and Sayan (*n* = 6), Scandinavia and Murmansk (*n* = 643), and Alps (*n* = 34). Spot diagrams show the occurrence of each colour pattern: grey circles show all sites represented in the sample, while black spots show those sites in which each colour pattern is recorded: unbanded pale tail (UP *n* = 329); yellow-banded pale tail (BP *n* = 1029); yellow-banded dark tail (BD *n* = 288). Map projection and other symbols as for Fig 1. Image created in ArcGIS using World_Shaded_Relief basemap which is Copyright 2014 Esri.

The principal colour patterns recorded for the subgenus *Alpinobombus* show three of the four possible combinations of the two states for the two characters we survey (Table 3, Fig 6): UP, unbanded pale tails; BP, banded pale tails, and BD, banded dark tails. The fourth state, entirely black (UD), is not recorded here, although one specimen of *B*. *balteatus* from Karaginskiy Island (Kamchatka, Russia) comes close, with only a narrow and incomplete yellow band on metasomal tergum 2. Fig 7 shows the distributions of the three principal colour patterns. The UP colour pattern is the most restricted geographically and is concentrated primarily in Europe; UP also occurs in isolated pockets in Russia and in North America but is unrecorded from Greenland. In contrast, the BP and BD patterns co-occur broadly, both taxonomically (Table 3) and geographically (Fig 7). The frequency of the BD colour pattern relative to BP colour pattern may increase towards the north (Fig 7). Nonetheless, the three principal colour-pattern groups are each shared by three of the nine prospective species (Table 3; *B*. *pyrrhopygus*, *B*. *polaris*, and *B*. *balteatus*), as well as being recorded from both the Old World and New World regions, as widespread polymorphisms. While there are often small differences between the most frequent patterns for each species, nonetheless in all cases it is possible to find specimens that resemble other species so closely that diagnosis by colour pattern is not always possible.

**Table 3:** Principal Colour Patterns for each Species of the Subgenus *Alpinobombus*.^a^

*alpinus*	*pyrrhopygus*	*polaris*	*balteatus*	*kirbiellus*	*neoboreus*	unnamed	*natvigi*	*hyperboreus*
	BD	BD	BD	BD	BD	BD	BD	BD
BP*	BP	BP	BP	BP	BP	BP		
UP	UP	UP	UP					

^a^Species are those recognised in Figs 2–4. Colour-pattern codes: UP, unbanded pale tail; BP, yellow-banded pale tail; BD, yellow-banded dark (black) tail. No unbanded dark tails have been recorded.

*For *B*. *alpinus*, males from the Alps often have a yellow-banded pattern although this is rare among females.

Pale tails are most often orange-red, but additional yellow- or white-tailed colour patterns of *B*. *balteatus* and *B*. *kirbiellus* are included.

**Fig 8:**
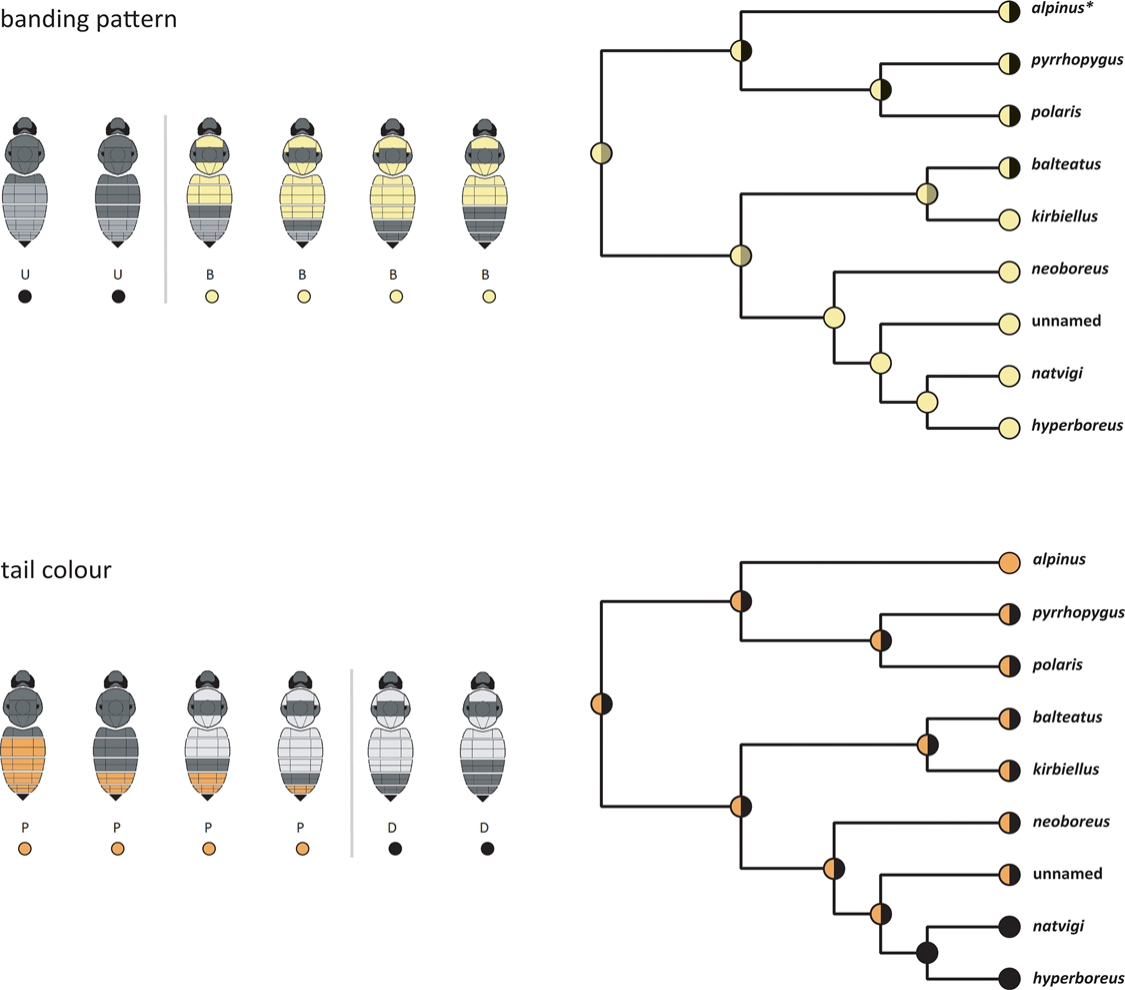
Evolution of Colour Patterns of the Subgenus *Alpinobombus*. Reconstruction of ancestral states by parsimony in Mesquite for two colour-pattern characters each with two states (Fig 6) among species based on the estimate of phylogeny in Fig 5, several of which are polymorphic and show both states for both characters. Above: banding colour pattern, with yellow spots showing yellow-banded (B) populations, black spots showing unbanded (U) populations, mixed yellow/black spots showing polymorphic populations, mixed yellow/grey spots showing uncertain polymorphic/monomorphic populations (*for *B*. *alpinus*, males from the Alps often have a yellow-banded pattern although this is rare among females). Below: tail colour pattern, with orange spots showing populations with pale (P: orange or white) hair on the tail, black spots showing populations with dark (D: black) hair on the tail, mixed orange/black spots showing polymorphic populations.

Reconstructions of the most parsimonious ancestral states for the two colour-pattern characters scored for the subgenus *Alpinobombus* are shown in Fig 8. For the banding pattern, Fig 8 supports either a root polymorphism with two subsequent reversals to a monomorphic banded pattern (to *kirbiellus* as well as to the ancestor of the group *neoboreus-hyperboreus*), or alternatively two forward changes to a polymorphic banded/unbanded pattern (to *balteatus* as well as to the ancestor of the group *alpinus-polaris*). For tail colour, Fig 8 supports a root ancestor with a polymorphism for tail colour. There is a reversal to a monomorphic pale tail for *alpinus* and a second forward change to a monomorphic black tail in the ancestor of the group *natvigi-hyperboreus*. Therefore we infer that both colour-pattern characters are likely to have had polymorphisms within the common ancestors of multiple species, which have then persisted in several of the descendent species as trans-species polymorphisms.

## Discussion

Bumblebees have long been used in discussions of the nature of species [8, 21, 23]. Many of the different ideas of what constitutes species [71] have been applied to bumblebees, including the biological species concept [72, 73] and specific-mate recognition systems [67, 74]. Among these ideas, pattern-based and process-based views have sometimes appeared to be irreconcilable [10, 75]. But more recently a consensus has emerged in favour of thinking of species generally as EILs, which provides a framework that is sufficiently broad to accommodate the many different criteria used to recognise species in practice, including both pattern-based and process-based approaches [1].

**Fig 9:**
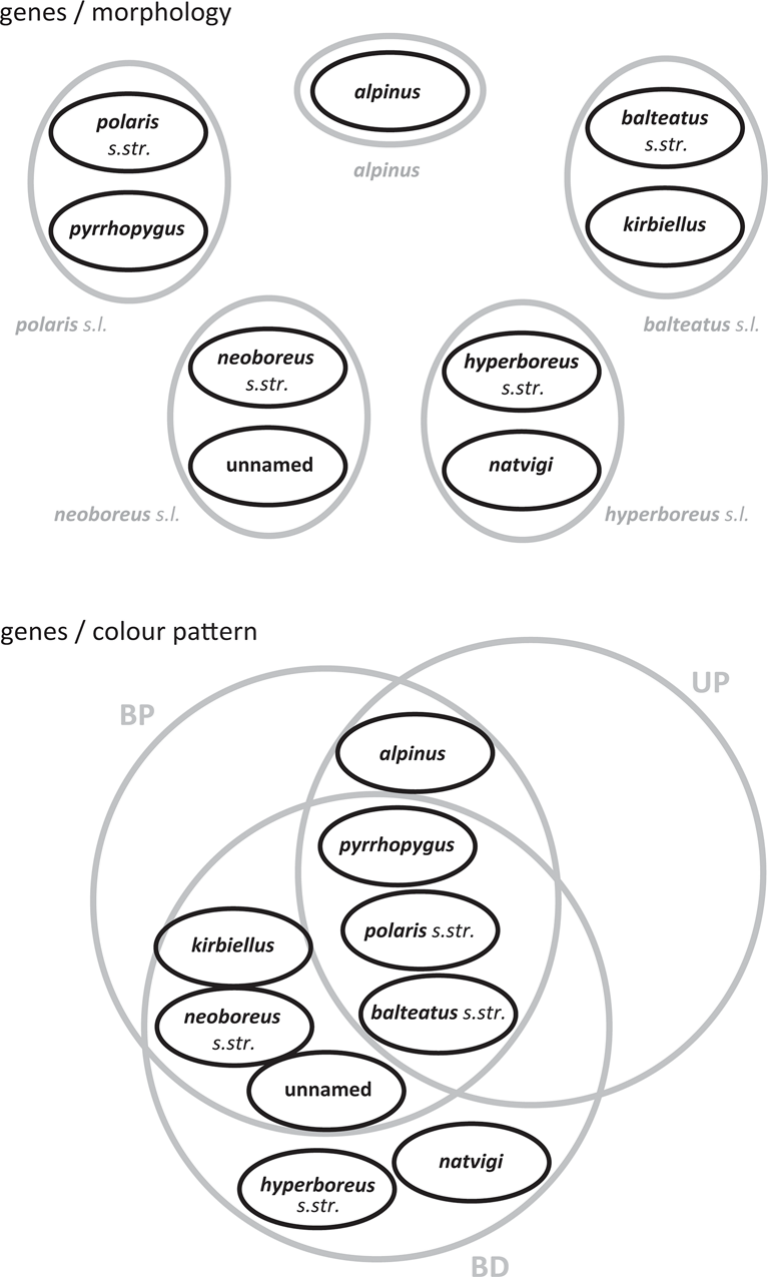
Disagreement among Groups of the Subgenus *Alpinobombus* Based on Three Character Sets. Above, set diagrams for the different but nested groups within *Alpinobombus* based on exoskeletal morphology (in grey) and based on genes (in black). Below, set diagrams for the different and mostly overlapping groups based on the colour patterns of the hair of the dorsum (in grey) and based on genes (in black). Areas of intersection of sets occupied by bumblebee samples are shown in grey. Abbreviations for colour patterns: BP, yellow-banded pale-tailed; UP, unbanded pale-tailed; and BD, yellow-banded dark-tailed.

The ability of the concept of species as EILs to accommodate multiple criteria has led to the idea of integrative taxonomy [2]. The integrative approach is based on the premise that the best way to discover species is to undertake multiple studies of the same set of individuals using different character sets and criteria, to compare the results, and then the best answer is found where different studies agree, i.e. through corroboration. Arguments that integration provides a ‘total evidence’ approach to bring out a shared underlying signal are less well founded, because the different character sets may have phylogenetic histories that are not shared [4]. The integrative approach has been applied previously for recognising bumblebee species [73, 76, 77]. However, our analysis of the subgenus *Alpinobombus* shows unusually weak agreement for bumblebees among groups from morphological, colour pattern, and genetic data. Morphology has been used in earlier studies to recognise five groups. Genetic data are used here to recognise nine groups, which although they are nested within the morphological groups, are mostly incongruent with them (Fig 9). Colour patterns can be used to recognise three principal groups, although most of the morphological or genetic groups overlap with more than one colour-pattern group (Fig 9). Within the framework of integrative taxonomy, this poor agreement among the groups supported by all three different character sets (the best that is achieved is nested sets: Fig 9) would require invoking special evolutionary explanations for each [2]. In this situation, the explanations might involve time lags between the evolution of some colour, genetic, and morphological characters [1] and this needs further investigation. This lack of congruency among groups based on different character sets may help to explain why previous taxonomic revisions have failed to agree on the number and identity of *Alpinobombus* species (Table 1).

Can we find other character sets that might support groups showing better agreement? Another approach for bumblebees considers characterising chemical extracts of male labial gland secretions. These extracts include sex-attractant or arrestant pheromones [77–80], which are expected to be important in specific-mate recognition systems [74]. These extracts can work well as indicators of species [79–82]. However, there is also evidence that within some bumblebee species there can be both divergence in these male secretions across different parts of a single species’ distribution, as well as divergence in the female preferences among these secretions [83]. In addition, and unfortunately for the case of *Alpinobombus*, when closely related bumblebee species have geographical distributions that do not overlap, at least sometimes the mate-recognition pheromones do not differ between these species [73]. We would anticipate that pheromones could be undifferentiated between the three recent east-west hemisphere pairs of sister species recognised in Figs 2–4, because these species are already prevented from interbreeding and given evolutionary independence through lack of gene flow, but caused in this case by ocean barriers (see below). Consequently there would be no selection for further reinforcement of barriers to interbreeding by causing divergence in sex pheromones.

Interbreeding and hybridisation are often seen as criteria central to species concepts [71–73]. Interbreeding is unlikely between the populations that we recognise here as sister species from their gene coalescents (e.g. between *B*. *polaris* and *B*. *pyrrhopygus*, between *B*. *balteatus* and *B*. *kirbiellus*, and between *B*. *hyperboreus* and *B*. *natvigi*) because interbreeding would have disrupted these coalescents. In each case, the distributions of these sister species are now separated so that they are prevented from dispersing and interbreeding by oceans, by 82 km at the Bering Strait and by >1000 km at the Greenland Sea. It is possible that bumblebees might be capable of dispersing across 82 km of sea, but it is unlikely that enough of them would arrive in a suitable physiological condition either to establish colonies or to mate successfully. But more persuasively, we see no evidence of dispersal, interbreeding, or introgression in this group from conflicts between the genetic and geographical data. In these cases, allopatric speciation is likely to have followed the repeated loss of the Bering land connection (which took place over as much as the last 5–7 Ma [84, 85]) according to dates estimated from our phylogenetic tree (Fig 5).

Another response to conflicting evidence has been to seek to accommodate the conflict within a hierarchy, by interpreting the broader groups as species and the narrower groups as subspecies [77, 86] (i.e. by choosing to reject the narrower groups as species). However, not only were gene-coalescent-based methods like GMYC specifically designed to discover species [49, 57, 60], not subspecies, but the justification for recognising subspecies is now far from universally accepted. Subspecies were introduced into bumblebee taxonomy originally as a typological concept, long before the biological species concept, and as a way of giving names to groups of individuals with differing colour patterns within a species [10]. Unfortunately, in practice the concept of subspecies has been applied with even less consistent meaning than the concept of species, to the point where subspecies have been considered to have hindered progress in taxonomy, evolutionary studies, and conservation [87–89]. For example, a recent review of the well-known bumblebee *B*. *terrestris* defines several subspecies from across Europe that include both discrete colour-pattern groups on islands, as well as dividing segments from more continuous colour-pattern clines across the continent [90]. It is unavoidable that dividing continuous clines is essentially arbitrary. This distinction between islands and the continent in colour-pattern variation is paralleled in evidence from normally highly variable DNA microsatellites, which also support many island groups as distinct, but which show no significant differentiation within the continental population [91]. We wish to avoid sometimes weakly-differentiated colour-pattern groups being over-interpreted in terms of (e.g.) ecology when this has not been justified by a thorough geographical analysis. At the same time, COI-barcode variation shows a single coalescent for the entire species [19] (see below regarding Corsica). To help avoid ambiguous differences in the meaning of subspecies in different cases, we agree that subspecies and trinomials should be replaced, either by direct informal descriptions of the particular colour patterns on which they are usually actually based and which they are used to indicate [67], or preferably by direct descriptions of the underlying genetic patterns from phylogeographic studies [30, 78].

Alternatively, when called upon to make taxonomic decisions in the face of conflicting evidence, we are prompted to consider which criterion within our framework is most appropriate and reliable for recognising species. If we accept the recent move from viewing species as morphotypes towards viewing them as EILs, then gene coalescents, which are very direct evidence of evolutionary independence, may be considered a special case as evidence for recognising species [1, 3, 49]. We still need to consider carefully whether sufficient conditions are likely to have been met to expect that fixed coalescents will have formed in each of these cases, for example whether populations are too large, or whether there is residual gene flow. We also accept that making the choice to view species differently (i.e. not as morphotypes) might alter the shape of taxonomy to some degree [2]. It has been argued that coalescence methods could reduce investigator-driven biases in species delimitation [49] and this should increase the precision in the groups recognised as species.

Both the GMYC and PTP methods have potential pitfalls and have to be applied with care [3, 59–61, 65]. For example, both are likely to split any samples isolated on trees by long terminal branches. These branches can become exaggerated as artefacts arising from a variety of causes, including sequencing errors [65], short sequences [59, 92], as well as unrepresentative sampling. For example, when samples from only Corsica and adjacent Europe were analysed with GMYC models, *B*. *xanthopus* Kriechbaumer was interpreted as an endemic Corsican bumblebee species separate from the mainland *B*. *terrestris* [86]. But when samples from throughout the known global distribution of *B*. *terrestris* were analysed [19], including samples from Madeira, the Canary Islands, North Africa, Europe, Russia, Iran, Central Asia, and from as far east as the indigenous eastern limit of the species in Mongolia, then the Corsican ‘*xanthopus’* samples were found to be part of the species *B*. *terrestris*, closely related to particular subgroups within that species (consistent with the results of an earlier analysis [91]). Sampling can never be ‘complete’, but best practice in morphological taxonomy [48] accepts that it is essential to sample from across the breadth of global ranges of all included taxa when revising any taxonomic group. The same principle holds when sampling genes, as we have attempted to do here (Fig 1). GMYC may also not work well when there has been extremely rapid speciation, either very recently or if populations were separated over a very short period long ago followed by a long slow divergence [59]. There is no reason to believe that these conditions apply in this case (Fig 5).

Some of our prospective species are currently known from few haplotypes, even though they represent larger samples. For the Old World *B*. *hyperboreus*, we have only two unique haplotypes shown in our trees (Figs 2 and 4), although these come from a sample of 13 Old World sequences. Six morphologically similar individuals from Greenland share the haplotype of *B*. *natvigi* from Canada, the oldest available name [39] for the New World sister species to the Old World species *B*. *hyperboreus*. In contrast, more material is needed to clarify the status of the ‘unnamed’ taxon, represented here by sequences from five individuals collected from high elevations in southern Alaska (Alaska range) and the Yukon (Saint Elias Mountains), sequences which differ from one another at two nucleotide positions. These sequences are unlikely to be paralogous nuclear pseudogenes of mtDNA, or ‘numts’ [93], because they have no indels, no stop codons, and have a higher variability with a strong bias towards high A/T frequency at codon position 3 [94]. Heteroplasmy has been found in bees [95], but we examined the trace files for these sequences and found no evidence of double or irregular peaks. Another possible explanation for distinguishing ‘unnamed’ might be incomplete lineage sorting [3], resulting in what might appear in our trees as paraphyly between *B*. *neoboreus* and the taxon ‘unnamed’. The counter argument is that the six *B*. *neoboreus* sequences (three of them sequenced twice, spanning a range from very pale to very dark specimens, with three unique haplotypes) differ from the five ‘unnamed’ sequences at 19 nucleotide positions (two of them causing amino acid changes at translation). This is sufficient for the GMYC analysis to recognise them as separate species. Independent corroborative support for the unnamed taxon as a separate species is shown by unique diagnostic base changes in the PEPCK gene (Table 2: 454 specimens have been examined for the two taxa combined, although most are too old for us to sequence). The biotic history of the Arctic and of the Beringian refuge may be complex [84, 96]: it is possible that *B*. *neoboreus* and the unnamed species could represent relict populations left from successive waves of range expansion and contraction following the cycles of climate change associated with the Pleistocene glaciations. For comparison, high elevations of the Yukon’s Kluane mountains are unusual for having outlying disjunct and genetically divergent populations of an otherwise arctic moth, *Gynaephora groenlandica* (Wocke) (Erebidae), and of the endemic arctic plants, *Oxytropis arctica* R. Br. (Fabaceae) and *Puccinellia vahliana* (Liebm.) Scrib. and Merr. (Poaceae), which have been interpreted as isolated relict populations [97].

Our results stand out compared with other revisionary studies of entire monophyletic bumblebee subgenera using genes [19, 76] in finding evidence that supports nearly twice as many species as had been recognised in morphology-based revisions. Most of the taxa of the subgenus *Alpinobombus* have been known for more than a century and only the one ‘unnamed’ species may not have been examined for previous revisions (Table 1). The other species recognised here for the first time from gene coalescents differ from the species splits in previous revisions (Table 1), which were based on differences in colour pattern. A large increase in species number is all the more surprising because the Arctic is expected to have relatively low biodiversity [98, 99]. Nonetheless, both the GMYC and PTP results (Figs 2–4) agree in separating Old World from New World sister populations for *B*. *polaris* / *B*. *pyrrhopygus*, *B*. *balteatus* / *B*. *kirbiellus*, and *B*. *hyperboreus* / *B*. *natvigi*. Discontinuity of populations as geographical disjunction is also a feature of morphologically cryptic species in other genetic studies of insects [100]. These patterns are at odds with ideas in some earlier revisions of the subgenus *Alpinobombus*, which had concluded that both sister species in some of these species pairs might occur either within the Old World (both of *B*. *polaris* and *B*. *pyrrhopygus*) [37] or within the New World (both of *B*. *balteatus* and *B*. *kirbiellus*) [36]. But, for *B*. *kirbiellus*, even the rare individuals with black hair on the side of the thorax (resembling many Old World *B*. *balteatus*) from as far north in the New World as Ellesmere Island do belong to *B*. *kirbiellus* according to our genetic results. Similarly, we infer that both banded and unbanded individuals belong to the Eurasian *B*. *pyrrhopygus*. The same nine species were also recognised by the ‘refined single-linkage’ (RESL) clustering procedure (single-linkage clustering followed by Markov clustering) that generates BOLD’s (boldsystems.org) Barcode Index Number (BIN) system from COI barcodes [101]. The RESL procedure is related to the empirical ‘barcoding gap’ idea. Consequently, while it is computationally fast, it lacks the theoretical justification of the coalescence-based approach, which is likely to cause results to diverge in some cases.

Unexpected and morphologically cryptic diversity has also been detected from genetic evidence in other groups of insects that had previously been studied intensively and had been considered taxonomically mature [100, 102]. Among the species recognised here but not in the most recent revisions, *B*. *kirbiellus* has been regarded as a separate species by just one reviser [36], although not with quite the same concept (the population from northern Canada was excluded). In contrast, *B*. *pyrrhopygus*, *B*. *natvigi*, and the unnamed species have no closely corresponding concepts of species in earlier revisions (Table 1). Intensity of sampling may be a factor in the discovery of the unnamed species, but for the others, the geographical breadth of this study is likely to be a key factor (along with the use of genetic data) allowing critical assessment of the variation.

Our results for the subgenus *Alpinobombus* are the first we know of for bumblebees to demonstrate support for close resemblance among parts of different species populations arising from shared ancestral polymorphisms (Fig 8). Many other cases of resemblance among more distantly related bumblebee species have been ascribed to evolutionary convergence [25, 26, 30]. Whereas spatial segregation (by latitude, longitude, or elevation) is characteristic of many of these convergent bumblebee-colour-pattern groups elsewhere in the world [26], we find broad spatial overlap between the individuals of *Alpinobombus* with the banded pale-tailed (BP) and the banded dark-tailed (BD) patterns. There may be some variation in the relative frequency of the two patterns with latitude (Fig 7). The unbanded pale-tailed (UP) pattern is more highly concentrated (longitudinally) in Europe, although it is also present in a few locations in both Asia and North America. The majority of species show more than one of these principal colour patterns, and one third of the species show all three (Table 3). This analysis uses our combined COI and PEPCK tree as an estimate of the species tree, which matches a tree for five of the species from five genes (including PEPCK but not COI) [32]. Therefore we feel it is reasonable at present to treat the combined COI and PEPCK tree as an estimate of the species tree. In any case, no estimate of phylogeny from more genes could alter the inference that ancestral polymorphisms are most likely to explain the pattern of extant polymorphisms within *Alpinobombus*, as long as a similar set of species is supported.

The principal weakness of our analysis of colour-pattern characters is that we do not yet know how colour patterns are inherited for bumblebees [103]. A diversity of possibilities for genes and molecular-development mechanisms is known to exist even among closely related species of flies within the genus *Drosophila* and among some mimetic butterflies, although within the butterfly genus *Heliconius* there is also evidence of a broadly conserved genetic basis for mimicry [104]. The simplest explanation for the pattern of states for the two characters of the subgenus *Alpinobombus* in Fig 8 is that in both cases divergence of the principal colour-pattern states preceded the divergence of many of the species. However, even though this result is more consistent with ancestral polymorphisms for both colour-pattern characters and this result is necessary in order to support the idea, it is not sufficient to prove the case. Ultimately proof will require identification of the genes governing these colour-pattern elements and the demonstration of homology of these genes among the species.

Why would an ancestral polymorphism persist, without one colour pattern becoming fixed in all populations by drift or selection? Local samples of the subgenus *Alpinobombus* we have examined do appear to show that local populations of a single species are often polymorphic (e.g. within *B*. *polaris* and Russian *B*. *pyrrhopygus*). Are these different colour patterns selectively neutral, or could there be heterogeneity in selection that actually favours the polymorphism, perhaps depending on temporal or spatial variation in thermal constraints or in the abundance of predators or ‘mimics’ in different microhabitats? Curiously, entirely black individuals of *Alpinobombus* are not present in our sample. Within Scandinavia, *B*. *balteatus* shows a tendency towards an increased frequency of darker colour patterns in the more southern mountains ([37]: her figure 69), which Løken associated with areas that have high humidity near the ground. Pekkarinen [41] recorded ‘almost completely black’ Scandinavian males of *B*. *balteatus* and described melanism in this species as ‘typical high-altitude or high-latitude’ melanism. Contrary to this assertion, the highest frequency of bumblebee species with many entirely black individuals world-wide occurs in the tropical lowlands ([26]; see also [105]). Our observations here show that for *Alpinobombus*, individuals from the highest latitudes (northern Greenland and Novaya Zemlya) are characterised by extensive yellow bands, although often with black tails, and there does appear to be some increase in the frequency of black-tailed individuals towards the north (Fig 7). The thermal properties of bumblebee colour patterns need further study [26].

## Conclusions

To examine gene coalescents as at least part of the evidence for discovering bumblebee species, studies should focus on obtaining: (a) homologous gene sequences; (b) comparable-length sequences; (c) sequences from multiple independent genes (which need to be rapidly evolving); and should (d) apply coalescence-based methods such as GMYC analysis. These aims supplement the more general sampling aims long recognised by morphological taxonomists [48] of the essential need to: (e) study a taxonomic group throughout its entire global range so as to include all constituent lineages or taxa; and (f) study many samples from throughout each constituent taxon’s range, both of which are just as important in genetic studies. Much more work is needed to elucidate the genetic control of bumblebee colour patterns and to assess the relative roles of ancestral polymorphisms and convergence in governing polymorphisms within species.

## Supporting Information

S1 File Table A. Depositories. Collections from which pinned material of the subgenus *Alpinobombus* has been examined.
(DOC)

S2 File Table A. Sequences. Sequenced samples of bumblebees (genus *Bombus*) of the subgenus *Alpinobombus* and outgroups with their accession numbers for GenBank (G) and the public project folder BBAL of the BOLD online database (B).
(DOC)
