# Supplementary material for: Genes Suggest Ancestral Colour Polymorphisms Are Shared across Morphologically Cryptic Species in Arctic Bumblebees
Source: PLoS One. 2015 Dec 10;10(12):e0144544. doi: 10.1371/journal.pone.0144544 (PMC4684343; doi:10.1371/journal.pone.0144544)
Supplement: S1 File — Collections from which pinned material of the subgenus Alpinobombus has been examined. (DOC) [file pone.0144544.s001.doc]

**Supporting Information**

**S1 Table A.** **Depositories**. Collections from which pinned specimens of the subgenus *Alpinobombus* have been examined.

| **Abbreviation** | **Collection** |
| --- | --- |
| AMNH | American Museum of Natural History, New York, USA |
| ANSP | Academy of Natural Sciences, Philadelphia, USA |
| BC | B. Cederberg research collection, Uppsala, Sweden |
| BP | B. Pedersen research collection, Copenhagen, Denmark |
| CNC | Canadian National Collection, Agriculture Canada, Ottawa, Canada |
| CR | C. Rasmussen research collection, Aarhus, Denmark |
| CS | C. Sheffield research collection, Regina, Canada |
| IBSSV | RAS Institute of Biology and Soil Science, Vladivostok, Russia |
| IEPN | RAS Institute of Ecological Problems of the North, Arkhangelsk, Russia |
| INHS | Illinois Natural History Survey, Champaign, USA |
| ISEAN | RAS Institute of Systematics and Ecology of Animals, Novosibirsk, Russia |
| JBME | University of Manitoba, J. B. Wallis Museum of Entomology, Winnipeg, Canada |
| JT | J. Thomson research collection, Toronto, Canada |
| KM | K. Martins research collection, Montreal, Canada |
| LACM | Los Angeles County Museum, Los Angeles, USA |
| LP | L. Packer research collection, Toronto, Canada |
| MB | M. Berezin research collection, Moscow, Russia |
| MNHU | Museum für Naturkunde an der Humboldt-Universität, Berlin, Germany |
| NHM | Natural History Museum, London, UK |
| NHRS | Swedish Museum of Natural History, Stockholm, Sweden |
| PW | P. Williams research collection, London, UK |
| PWRC | Patuxent Wildlife Research Center, Laurel, USA |
| RMBL | Rocky Mountain Biological Laboratory, Crested Butte, USA |
| ROM | Royal Ontario Museum, Toronto, Canada |
| RUP | Rutgers University, Piscataway, USA |
| UAF | University of Alaska Fairbanks, Fairbanks, USA |
| UCB | University of California, Essig Museum of Entomology, Berkeley, USA |
| UCD | University of California, Bohart Museum of Entomology, Davis, USA |
| UCMNH | University of Colorado Museum of Natural History, Boulder, USA |
| UCR | University of California, Riverside, USA |
| UOG | University of Guelph, Guelph, Canada |
| UOK | University of Kansas, Snow Entomological Museum Collection, Lawrence, USA |
| UUL | University of Utah, Logan, USA |
| USNM | US National Museum of Natural History, Washington DC, USA |
| YPM | Yale University, Peabody Museum of Natural History, New Haven, USA |
| ZIL | Zoological Institute, University of Lund, Lund, Sweden |
| ZISP | RAS Zoological Institute, St Petersburg, Russia |
| ZMMU | Zoological Museum of the Moscow State University, Moscow, Russia |
| ZMO | Zoological Museum, University of Oslo, Oslo, Norway |
| ZMU | Museum of Evolution, Uppsala, Sweden |
