# Supplementary material for: Genes Suggest Ancestral Colour Polymorphisms Are Shared across Morphologically Cryptic Species in Arctic Bumblebees
Source: PLoS One. 2015 Dec 10;10(12):e0144544. doi: 10.1371/journal.pone.0144544 (PMC4684343; doi:10.1371/journal.pone.0144544)
Supplement: S2 File — Sequenced samples of bumblebees (genus Bombus) of the subgenus Alpinobombus and outgroups with their accession numbers for GenBank (G) and the public project folder BBAL of the BOLD online database (B). (DOC) [file pone.0144544.s002.doc]

**Supporting Information**

**S2 Table A.** **Sequences**. Sequenced samples of bumblebees (genus *Bombus*) of the subgenus *Alpinobombus* and outgroups with their accession numbers for GenBank (G) and the public project folder BBAL of the BOLD online database (B).

| **Species** | **Database #** | **Sample location** | **Collector** | **Gene-sequence #** |
| --- | --- | --- | --- | --- |
| *cryptarum* (Fabricius) | ‒ | Turkey | P Rasmont et al. | PEPCK G:EF050855 |
| *ignitus* Smith | ‒ | China | S Cameron et al. | PEPCK G:EF050866 |
| *rufocinctus* Cresson | ‒ | Canada | A Scholl | PEPCK G:EF050892 |
| *terrestris* (Linnaeus) | ‒ | Italy | S Cameron et al. | PEPCK G:EF050865 |
| *vagans* Smith | ‒ | USA | J Whitfield | PEPCK G:EF050854 |
| *alpinus* (Linnaeus) | ‒ | Austria | S Cameron et al. | PEPCK G:EF050871 |
| *alpinus* (Linnaeus) | 114 | Sweden | P Bina | PEPCK G:KT954148 |
| *balteatus* Dahlbom | ‒ | Sweden | B Cederberg | PEPCK G:EF050870 |
| *balteatus* Dahlbom | 3836 | Norway | F Ødegaard | PEPCK G:KT954152 |
| *pyrrhopygus* Friese | ‒ | Sweden | H Elmquist | PEPCK G:EF050872 |
| *pyrrhopygus* Friese | 3825 | Norway | F Ødegaard | PEPCK G:KT954149 |
| *pyrrhopygus* Friese | 3832 | Norway | F Ødegaard | PEPCK G:KT954150 |
| *hyperboreus* Schönherr | ‒ | Sweden | B Cederberg | PEPCK G:EF050868 |
| *hyperboreus* Schönherr | 3820 | Norway | F Ødegaard | PEPCK G:KT954158 |
| *kirbiellus* Dahlbom | 65 | USA | ‒ | PEPCK G:KP901105 |
| *kirbiellus* Dahlbom | 3533 | Canada | ‒ | PEPCK G:KT954153 |
| *kirbiellus* Dahlbom | 3534 | Canada | ‒ | PEPCK G:KT954154 |
| *natvigi* Richards | 3546 | Canada | ‒ | PEPCK G:KP901106 |
| *natvigi* Richards | 3516 | Canada | ‒ | PEPCK G:KT954157 |
| *neoboreus* Sladen | ‒ | USA | A Scholl | PEPCK G:EF050869 |
| *neoboreus* Sladen | 68 | USA | ‒ | PEPCK G:KT954155 |
| *polaris* Curtis | 44 | Greenland | C Rasmussen | PEPCK G:KP901107 |
| *polaris* Curtis | 4395 | USA | D Sikes | PEPCK G:KT954151 |
| unnamed | 16 | Canada | ‒ | PEPCK G:KP901108 |
| unnamed | 4392 | USA | J Rykken | PEPCK G:KT954156 |
| *cryptarum* (Fabricius) | 113 | UK | ‒ | COI G: KP849482 |
| *ignitus* Smith | ‒ | China | J An | COI G:JQ843431 |
| *rufocinctus* Cresson | ‒ | Canada | ‒ | COI B:0289G06 |
| *terrestris* (Linnaeus) | ‒ | Switzerland | R Schmid-Hempel | COI G:JQ843641 |
| *vagans* Smith | ‒ | USA | C Scully | COI B:3746B07 |
| *alpinus* (Linnaeus) | 4246 | Norway | J Gjershaug | COI B:BOMN166 |
| *alpinus* (Linnaeus) | 114 | Sweden | P Bina | COI B:20945A02 |
| *alpinus* (Linnaeus) | 1 | Switzerland | P Williams | COI B:6873F07 |
| *alpinus* (Linnaeus) | 2 | Italy | C Schmid-Egger | COI G:HQ948121 |
| *pyrrhopygus* Friese | 3832 | Norway | F Ødegaard | COI B:20945H02 |
| *pyrrhopygus* Friese | 553 | Russia | M Berezin | COI B:20945B11 |
| *pyrrhopygus* Friese | 3828 | Norway | F Ødegaard | COI B:20945H07 |
| *pyrrhopygus* Friese | 4247 | Norway | F Ødegaard | COI B:NOAPI563 |
| *pyrrhopygus* Friese | 3831 | Norway | F Ødegaard | COI B:20945G04 |
| *pyrrhopygus* Friese | 3827 | Norway | F Ødegaard | COI B:20945F06 |
| *pyrrhopygus* Friese | 4237 | Norway | F Ødegaard | COI B:NOAPI641 |
| *pyrrhopygus* Friese | 3834 | Norway | F Ødegaard | COI B:20945H03 |
| *polaris* Curtis | 29 | Canada | M Hannan | COI G:JX829309 |
| *polaris* Curtis | 24 | Canada | M Hannan | COI G:JX831851 |
| *polaris* Curtis | 28 | Canada | M Hannan | COI G:JX832483 |
| *polaris* Curtis | 26 | Canada | M Hannan | COI G:JX830137 |
| *polaris* Curtis | 20 | Canada | L Packer | COI B:6735D01 |
| *polaris* Curtis | 97 | Canada | ‒ | COI B:15289A02 |
| *polaris* Curtis | 110 | Canada | ‒ | COI B:9863C04 |
| *polaris* Curtis | 57 | Greenland | C Rasmussen | COI B:6879D06 |
| *polaris* Curtis | 53 | Greenland | J Mosbacher | COI B:6879D02 |
| *polaris* Curtis | 56 | Greenland | C Rasmussen | COI B:6879D05 |
| *balteatus* Dahlbom | 3843 | Norway | F Ødegaard | COI B:20945G12 |
| *balteatus* Dahlbom | 4248 | Norway | F Ødegaard | COI B:NOAPI567 |
| *balteatus* Dahlbom | 537 | Russia | M Berezin | COI B:20945B07 |
| *balteatus* Dahlbom | 538 | Russia | M Berezin | COI B:20945D07 |
| *balteatus* Dahlbom | 539 | Russia | M Berezin | COI B:20945B06 |
| *balteatus* Dahlbom | 3 | Mongolia | J Gelhaus | COI B:1550F10 |
| *kirbiellus* Dahlbom | 7 | USA | B Thompson | COI B:3760H06 |
| *kirbiellus* Dahlbom | 95 | Canada | J Mahalingam | COI B:LRBBC2186 |
| *kirbiellus* Dahlbom | 94 | Canada | J Mahalingam | COI B:LRBBC2185 |
| *kirbiellus* Dahlbom | 65 | USA | ‒ | COI B:6879E12 |
| *kirbiellus* Dahlbom | 4 | Canada | R Layberry | COI B:3759F08 |
| *neoboreus* Sladen | 13 | Canada | S Cannings | COI B:15289C04 |
| *neoboreus* Sladen | 68 | USA | ‒ | COI B:6879F03 |
| *neoboreus* Sladen | 92 | Canada | A Gunn | COI B:15289B05 |
| unnamed | 16 | Canada | S Cannings | COI B:6717E06 |
| unnamed | 17 | Canada | S Cannings | COI B:6717F04 |
| *hyperboreus* Schönherr | 4236 | Norway | F Ødegaard | COI B:NOAPI569 |
| *hyperboreus* Schönherr | 533 | Russia | M Berezin | COI B:20945D06 |
| *natvigi* Richards | 82 | Canada | ‒ | COI B:12076E01 |
| *natvigi* Richards | 87 | Canada | ‒ | COI B:12076F05 |
| *natvigi* Richards | 83 | Canada | ‒ | COI B:12076E02 |
| *natvigi* Richards | 84 | Canada | ‒ | COI B:12076E03 |
| *natvigi* Richards | 105 | Canada | ‒ | COI B:9863C06 |
| *natvigi* Richards | 81 | USA | J Fox | COI B:6879G04 |
|  |  |  |  |  |
